# Socio-economic vulnerabilities and HIV: Drivers of transactional sex among female bar workers in Yaoundé, Cameroon

**DOI:** 10.1371/journal.pone.0198853

**Published:** 2018-06-18

**Authors:** Derick Akompab Akoku, Mbah Abena Tihnje, Thomas Achombwom Vukugah, Elvis Enowbeyang Tarkang, Robinson Enow Mbu

**Affiliations:** 1 Community Research and Training Institute, Yaoundé, Cameroon; 2 Ministry of Public Health, Yaoundé, Cameroon; 3 School of Public Health, University of Health and Allied Sciences, Ho, Ghana; 4 Faculty of Medicine and Biomedical Sciences, University of Yaoundé 1, Yaoundé, Cameroon; London School of Hygiene and Tropical Medicine, UNITED KINGDOM

## Abstract

**Introduction:**

The purpose of this study was to examine the relationship between socio-demographic characteristics, risky sexual behaviour, alcohol use and transactional sex among female bar workers in Yaounde, Cameroon.

**Materials and methods:**

A cross-sectional survey was conducted among a representative sample of 410 female bar workers, recruited through a modified version of venue-based cluster sampling technique from May to June 2017. Transactional sex was defined as having received money/gifts in exchange for sex with any sexual partner in the past 12 months. Logistic regression models were performed to identify the factors associated with transactional sex. The level of statistical significance was set at p< = 0.05.

**Results:**

About 14.9% (n = 61) of respondents reported to have engaged in transactional sex, 83.7% (n = 338) had multiple sexual partners at the time of the study, 14.4% (n = 55) had sex with one or more of their male customers in the past 6 months. Almost 73.4% (n = 301) reported alcohol use. Of these, 37.2% (n = 112) were frequent alcohol consumers. About 17.6% (n = 72) reported to have had unprotected sex under the influence of alcohol in the past 6 months. Multivariate logistic regression analysis showed that those who engaged in transactional sex were more likely to have had sex with a male customer in the past 6 months (aOR = 7.34; 95% CI, 3.63–16.98), had sex under the influence of alcohol in the past 6 months (aOR = 2.42; 95% CI, 1.18–4.96) and frequent alcohol consumers (aOR = 2.06; 95%CI, 1.04–4.10). Respondents who had their last sexual intercourse 4 weeks or more prior to the study (aOR = 0.26; 95% CI, 0.08–0.84) were less likely to have engaged in transactional sex.

**Conclusions:**

Our study concludes that female bar workers are exposed to male customers and engage in risky sexual practices including transaction sex. Most of them also consume alcohol which increases their risk of HIV and STI acquisition. They are a high-risk group that need to be targeted with HIV prevention interventions.

## Introduction

HIV/AIDS and other sexually transmitted infections (STI) still pose a significant public health challenge in Cameroon. With a population of about 23 million inhabitants [1], the country has an estimated HIV prevalence of 4.3% among adults aged 15–49 years [2]. Young girls and women continue to be disproportionately affected by HIV than their male counterparts. The prevalence of HIV was reported to be almost twice as high among women (5.6%) compared to men (2.9%)[2]. Among women, the HIV prevalence is higher among those living in urban areas (6.4%) compared to those living in rural areas (4.6%)[2]. Generally, many factors have been reported to increase women’s vulnerability to HIV including biological, social, behavioural, cultural, economic and structural [3–5].

Transactional sex, defined as sex in exchange for money or material gifts [6, 7] has been demonstrated to increase women’s vulnerability to HIV [6, 8, 9]. Women have been reported to engage in transactional sex for a variety of reasons including economic, material and social gains; and access to basic necessities such as food, shelter, protection, clothing, hygiene requirements and school necessities [10–13]. Studies have shown that women who engage in transactional sex are at an elevated risk of HIV acquisition [8, 9]. Transactional sex has also been associated with risky sexual behaviours including age-disparage sex, multiple concurrent sexual partnerships, unprotected sex and intimate partner violence [9, 14–16]. Studies have reported that many women in sub-Saharan Africa (SSA) who engage in transactional sex do not self-identify themselves as sex workers due to the stigmatising connotation attached to sex work [6, 13, 16].

Although HIV vulnerability and transactional sex has been extensively studied among many women’s group including female sex workers [17], female bar workers (FBWs) represent a subgroup of women whose HIV vulnerability has received little attention in scholarly research. FBWs are women who work in the informal alcohol industry, promoting the sale of beer and alcoholic drinks to customers in bars, karaoke, beer parlours and casinos. These women are encouraged by their employers to socialize with male customers to increase the sale and consumption of beer [18]. Due to their low income, FBWs are forced to sell sex on a regular basis or occasionally to their male customers to supplement their income [19, 20]. This explains why these women are sometimes classified as indirect sex workers [20–22].

Studies have established a positive association between alcohol consumption and risky sexual behaviours [23, 24] including transactional sex among the general population [25]. Previous research conducted among FBWs in Cambodia showed that these women frequently consume alcohol [26] and some are invited by their male customers to drink with them. These FBWs are forced to drink alcohol upon invitation to increase the sale of beer and receive tips from their male customers [19]. In certain situations, they may have sex with their male customers after consuming alcohol which increases their HIV vulnerability. In Cambodia, FBWs have been reported to have unprotected sex with their male customers and this sometimes occurred under the influence of alcohol [27].

In Cameroon, alcohol consumption is a common practice in many socio-cultural milieu. There are many FBWs (although their estimated population is unknown) promoting beer consumption in bars, karaoke, beer parlours and casinos in the country. However, there has been no research conducted among this subgroup of women to examine their HIV vulnerability. As a result, little is known about the extent to which these women are vulnerable to HIV and STI infection and what factors contribute to their risky sexual practices. Furthermore, while the relationship between alcohol use and risky sexual behaviour among women in the general population has been established [7, 25, 28], this association has not been investigated among this subgroup of women. The lack of empirical data has made it difficult to determine their level of HIV and STI vulnerability and this perhaps explains why there has been limited HIV prevention interventions targeted at this subgroup of women in Cameroon. Rather, most HIV prevention interventions have targeted high risk groups of women like female sex workers due to evidence of their vulnerability to HIV and STI [29].

The objective of this study was to examine the association between socio-demographic characteristics, alcohol use and transactional sex among FBWs in Yaoundé, Cameroon. Understanding these relationships will fill the knowledge gaps and provide the evidence that will inform HIV prevention strategies and interventions among this subgroup of women in the country.

## Materials and methods

### Study design and setting

The study was a cross-sectional population-based study conducted in Yaounde, the political capital of Cameroon. Yaounde has an estimated population of 2.4 million inhabitants [30] and is made up of seven administrative zones. It lies in the centre of the nation at an elevation of about 750 metres (2,500 ft) above sea level. It is the centre of administration, service industry and commerce, and a key point for road, railway and air travels. Yaounde is an ethnically, linguistically, and religiously diverse city attracting individuals from all corners of the country. It is a bustling city and its inhabitants include people from all walks of life. Yaounde has a brewery industry and many bars, restaurants, karaoke, casinos and other entertainment venues.

### Study population and sampling procedure

The study population was FBWs promoting the sale of beer and other alcoholic drinks in bars, beer parlours, karaoke and casinos. The minimum sample size was derived from the formula: n = [z^2^ x p (1-p)] DEFF / [m^2^]; where n = the minimum required sample size for the study; z = the standard deviation for a 2-tailed test at 95% confidence level (1.96); p (4.3%) = the estimated population parametre; DEFF (2.0) the design effect, m = the margin of error (3%) and an anticipated non-response rate of 12%. The minimum estimated sample size for the study was 399 respondents. A modified version of venue-based cluster sampling technique was used to sample participants. In each of the seven administrative zones of Yaounde, we randomly selected city blocks (neighbourhoods) with the probability of selection based on the population size (Galway, Bell et al. 2012) of that administrative zone using census data from the National Institute of Statistics. In each selected city block (neighbourhood), we identified entertainment venues (bars, beer parlours, karaoke etc). We also conducted key-informant interviews to obtain more information on the venues where FBWs could be located. A list of entertainment venues (where FBWs work) in each selected city block was established and this constituted the sampling frame for the current study. From the list of entertainment venues in each selected city block, a random sample of entertainment venues was visited by field teams. FBWs working in the selected venues were the tertiary sampling unit.

### Data collection

Data were collected from May to June 2017 through face-to-face interviews by trained interviewers. Owners of venues selected were approached, informed about the study and their permission/collaboration sought to collect data from their sites. Once permission was granted, interviewers approached the participants and determined their eligibility for participation. Participants were eligible if they were aged at least 21 years at the time of the study, had worked as a FBW for at least 3 months and provided written informed consent to participate in the study. Those who did not express their willingness to participate and refused to consent were excluded from the study. When a participant refused to participate, interviewers moved to the next participant (if there were more than one FBW at the venue) or the next entertainment venue based on the sampling list. Those who refused to participate indicated lack of time, lack of interest in the study or simply because they did not want to participate. Interviewer-administered paper-based questionnaires (S1 Questionnaire) were completed in a quiet and private location to ensure confidentiality. Participants could choose not to answer certain questions in the questionnaire. Data were collected during non-peak hours (mornings and early afternoon) to enable FBWs fully participate. This was because FBWs are usually busy during peak hours (late afternoons and evenings) promoting and serving beer to customers.

### Measures

#### Outcome variable

The primary outcome variable was transactional sex. Transactional sex was defined as having had sex in exchange for money or gift in the past 12 months; and this was assessed by asking participants: “Have you had sex in exchange for money or gift in the past 12 months? The response options were “No” and “Yes”.

#### Explanatory variables

The questionnaire collected data on: 1) socio-demographic characteristics, 2) sexual behaviour, and 3) alcohol use. *Socio-demographic* characteristics included age, marital status, educational attainment, monthly income, other sources of income, living arrangements, number of household members and duration of work as a FBW.

*Sexual behaviour* was assessed by collecting data on: number of life time sexual partners, age at sexual debut, sex with a male customer in the past 6 months, multiple sexual partnership, last time they had sexual intercourse and unprotected sex under the influence of alcohol in the past 6 months.

*Alcohol use* was assessed by asking participants: *“Do you consume alcohol*?*” The response options were “No” and “Yes”*. Those who indicated “Yes” were asked: *“During the past one month*, *on how many days did you have at least one drink of alcohol*?*”* The response options (in days) were: 1–2, 3–5, 6–9, 10–19, 20–29, and all the 30 days. Those who reported to have consumed alcohol 10 days and above were classified as “Frequent” alcohol consumers while those who reported to have consumed alcohol below 10 days in the last 30 days were classified as “Non-frequent” alcohol consumers.

### Ethical considerations

Ethical clearance was obtained from the Cameroon National Research Ethics Committee for Human Health (No: 2016/840/CE/CNERSH/SP). Administrative authorization was also obtained from the Centre Regional Delegation for Public Health to conduct the study in Yaounde. Participation in this study was voluntary, and written informed consent was obtained from all participants who expressed interest to participate. Study participants had an opportunity to refuse or to opt-out of the study at any time. Privacy was protected as face-to-face interviews were conducted in a private location. Confidentiality was ensured as no personal identifiers were collected.

### Statistical analysis

To account for the unequal sampling probabilities, data (S1 Study Dataset) were weighted prior to analysis. A sampling weight for each participant was calculated as the inverse of the probability of selection of that participant taking into account each stage of the sampling process. Descriptive analyses such as frequencies and proportions, median and interquartile range were performed to examine participants’ characteristics. Pearson's chi-square tests were used to identify associations between socio-demographic characteristics, sexual behaviour, alcohol consumption and transactional sex. Student’s t-test was conducted to assess differences between the continuous variable (age at sexual debut) and the dependent variable (transactional sex). Variables that were significantly associated with transactional sex at p-value <0.1 were considered candidates for logistic regression analyses.

Univariate and multivariate logistic regression analyses were performed to estimate the strength of the associations between the dependent variable (transactional sex) and the significant independent variables from the bivariate analyses. In the multivariate model controlling for potential confounders, there was no evidence of multicollinearity among the independent variables. Adjusted odds ratio (aOR) with 95% confidence levels (CI) were used to quantify the strength of the association. The statistical significance for all tests was set at p< = 0.05. Data were analysed using STATA version 13.0 (StataCorp, College Station, Texas, USA).

## Results

### Socio-demographic characteristics by transactional sex

A total of 506 respondents were screened and eligible for the study. Of these, 415 agreed to participate (response rate = 82%). However, due to missing data, 5 questionnaires were eliminated leaving 410 for analysis. Table 1 shows respondents’ socio-demographic characteristics by transactional sex. The median age of the 410 study respondents was 29 years (IQR, 25–34). Over half (58.8%) were aged 21–30 years, most (78.5%) were never married, 75.9% attained education below high school, and 67.8% had a monthly income of less than 50,000 FCFA (< 89 US $). The majority (86.6%) had no other source of income apart from what they earn as a FBW. Those who had other sources of income were significantly more likely to have engaged in transactional sex in the past 12 months than those without other sources of income (23.6% vs 13.5%, p = 0.034). About 80.9% were living with someone; those who reported to have engaged in transactional sex where significantly more likely to be living alone compared to those living with others (21.8% vs 13.3%, p = 0.039). An estimated 40.4% were living with 4–6 household members, and almost half (48.1%) had worked as a FBW for at least a year.

**Table 1:** Socio-demographic characteristics by transactional sex.

Characteristics	Total sample n (%)	Transactional sex in the past 12 months n(%)		P-value
		No	Yes	
Sample size	410 (100)	349 (85.1)	61 (14.9)	
**Age group**				0.130
21–30	239 (58.3)	205 (85.8)	34 (14.2)	
31–40	137 (33.4)	112 (81.8)	25 (18.3)	
41+	34 (8.3)	32 (94.1)	2 (5.9)	
**Marital Status**				0.636
Never Married	322 (78.5)	272 (84.5)	50 (15.5)	
Married	66 (16.1)	57 (86.4)	9 (13.6)	
Divorce/Separated/Widowed	22 (5.4)	20 (90.9)	2 (9.1)	
**Educational attainment**				0.543
Below high school	311 (75.8)	263 (84.6)	48 (15.4)	
High school and above	99 (24.2)	86 (86.9)	13 (13.1)	
**Monthly Income**^1^				0.396
<50,000 FCFA (approx. US$ 89)	278 (67.8)	234 (84.2)	44 (15.8)	
> = 50,000 FCFA	132 (32.2)	115 (87.1)	17 (12.9)	
**Has other sources of income**				0.034
No	355 (86.6)	307 (86.5)	48 (13.5)	
Yes	55 (13.4)	42 (76.4)	13 (23.6)	
**Current living arrangements**				0.039
Alone	78 (19.1)	61 (78.2)	17 (21.8)	
With someone	332 (80.9)	288 (86.8)	44 (13.3)	
**Number of household members‡ (n = 322)**				0.837
1–3	127 (39.4)	111 (87.4)	16 (12.6)	
4–6	130 (40.4)	113 (86.9)	17 (13.1)	
7 or more	72 (20.2)	55 (84.6)	10 (15.4)	
**Work duration as a FBW**				0.611
3–12 months	213 (51.9)	183 (85.9)	30 (14.1)	
13 months and above	197 (48.1)	166 (84.3)	31 (15.7)	

**Notes:** Numbers are unweighted, percentages are weighted.

^1^Exchange rate of 1US$ = 558 FCFA as at August 2017. The minimum wage in Cameroon as at August 2017 was 36,270 FCFA.

P- values were calculated from Chi-square tests

‡Only for those who live with someone

### Sexual behaviour and alcohol use by transactional sex

Table 2 shows associations between sexual behaviour and alcohol use by transactional sex in the past 12 months. About 77.1% of FBWs reported to have had less than 10 life time sexual partners and a minority (5.6%) had 20 or more life time sexual partners. Among this small group with 20 or more partners, a high percentage (39.1%) reported recent transactional sex. The median age of sexual debut for all respondents was 18 years (IQR = 16–19). The median age of those who reported to have engaged in transactional sex was slightly lower than those who did not engage in transactional sex (17 yrs vs 18 yrs, p = 0.050).

**Table 2:** Sexual behaviour and alcohol use by transactional sex.

Characteristics	Total sample n(%)	Transactional sex in the past 12 months n (%)		P-value
		No	Yes	
**Number of life time sexual partners**				0.001
0–9	316 (77.1)	274 (86.7)	42 (13.3)	
10–19	71 (17.3)	61 (85.9)	10 (14.1)	
20+	23 (5.6)	14 (60.9)	9 (39.1)	
**Age at sexual debut (median/IQR)**	18 (16–19)	18 (16–19)	17 (15–18)	0.050
**Had sex with a male customer in the past 6 months**				
No	355 (86.6)	319 (89.9)	36 (10.1)	<0.001
Yes	55 (14.4)	30 (54.6)	25 (45.4)	
**Has multiple sexual partners**				0.227
No	66 (16.3)	59 (89.4)	7 (10.6)	
Yes	338 (83.7)	284 (84.1)	54 (19.9)	
**Last sexual intercourse**				0.015
< 1 week ago	193 (47.1)	155 (80.3)	38 (19.7)	
1–2 weeks ago	86 (20.9)	73 (84.9)	13 (15.1)	
3–4 weeks ago	37 (9.0)	34 (91.9)	3 (8.1)	
5 weeks or more ago	94 (22.9)	87 (92.6)	7 (7.4)	
**Used a condom during last sex**				0.930
No	264 (64.4)	225 (85.2)	39 (14.8)	
Yes	146 (35.6)	124 (84.9)	22 (15.1)	
**Had unprotected sex under the influence of alcohol in the past 6 months**				0.001
No	338 (82.4)	72.2	12.9	
Yes	72 (17.6)	10.2	46.3	
**Consumes alcohol**				0.004
No	109 (26.6)	93 (85.3)	16 (14.7)	
Yes	301 (73.4)	256 (85.1)	45 (14.9)	
**Frequency of alcohol consumption**				
Non-frequent	189 (62.8)	171 (90.5)	18 (9.5)	<0.001
Frequent	112 (37.2)	86 (76.8)	26 (23.2)	

**Notes:** IQR = Interquartile range.

Numbers are unweighted, percentages are weighted

P- values were calculated from Chi-square tests except for age at sexual debut which was calculated with t-test.

About 14.4% of the FBWs indicated that they had sex with a male customer in the past 6 months. Those who reported to have had sex with a male customer were significantly more likely to have engaged in transactional sex in the past 12 months (45.4% vs 10.1%, p = <0.001) compared to those who did not have sex with a male customer. Most (83.7%) of the FBWs indicated that they had more than one sexual partner at the time of the study. Nearly half (47.1%) had their last sexual intercourse within a week prior to the study. Those who had sex less than one week prior to the study were more likely to have engaged in transactional sex than those who had their last sexual intercourse five or more weeks prior to the study (19.7% vs 7.4%, p = 0.001). Overall, only 35.6% of respondents used a condom during their last sexual intercourse. Respondents who used a condom during their last sexual intercourse where more likely to have engaged in transactional sex than those who did not use a condom (15.1% vs 14.8%). The majority (73.4%) of participants were alcohol consumers. Among those who consume alcohol, more than half (62.8%) were classified as “Non-frequent” alcohol consumers. Nevertheless, “Frequent” consumers of alcohol were more likely to have engaged in transactional sex in the past 12 months compared to “Non-frequent” consumers, (23.2% vs 9.5%, p<0.001).

### Predictors of transactional sex

Table 3 shows the predictors of transactional sex in the past 12 months among FBWs. In weighted univariate analysis, FBWs with other sources of income (OR = 1.97;95%CI = 1.04–3.76, p = 0.37), who had 20 or more life time sexual partners (OR = 4.19;95%CI,1.83–9.63, p = 0.001), who had sex with a male customer in the past 6 months (OR = 7.38; 95%CI = 4.11–13.26, p<0.001), who had sex under the influence of alcohol in the past 6 months (OR = 2.52; 95%CI, 1.14–4.46,p = 0.001) and who were “Frequent” alcohol consumers(OR = 2.87;95% CI,1.56–5.27,p = 0.001) were more likely to have engaged in transactional sex in the past 12 months. Those who were living with others (OR = 0.54; 95%CI, 0.31–0.97, p = 0.041) and who had sex more than a month prior to the study (OR = 0.33; 95%CI, 0.15–0.72, p = 0.005) were less likely to have engaged in transactional sex in the past 12 months.

**Table 3:** Logistic regression models for predictors of transactional sex.

Variables	Weighted univariate		Weighted multivariate	
	OR (95% CI)	P-value	aOR (95% CI)	P-value
**Has other sources of income**				
No	1.0		1.0	
Yes	1.97 (1.04–3.76)	0.037	2.07 (0.94–4.59)	0.070
**Current living arrangements**				
Alone	1.0		1.0	
With someone	0.54 (0.31–0.97)	0.041	0.51 (0.23–1.09)	0.083
**Number of life time sexual partners**				
0–9	1.0		1.0	
10–19	1.06 (0.53–2.13)	0.848	0.80 (0.36–1.78)	0.592
20+	4.19 (1.83–9.63)	0.001	2.92 (0.69–12.0)	0.142
**Age at sexual debut**	0.89 (0.78–1.02)	0.095	1.05 (0.85–1.29)	0.625
**Had sex with a male customer in the past 6 months**				
No	1.0		1.0	
Yes	7.38 (4.11–13.26)	<0.001	7.84 (3.63–16.98)	<0.001
**Last sexual intercourse**				
< 1 week ago	1.0		1.0	
1–2 weeks ago	0.72 (0.38–1.37)	0.235	1.34 (0.56–3.18)	0.502
3–4 weeks ago	0.35 (0.11–1.13)	0.079	1.29 (0.36–4.66)	0.686
More than 4 weeks ago	0.33 (0.15–0.72)	0.005	0.26 (0.08–0.84)	0.024
**Used a condom during last sex**				
No	1.0			
Yes	1.02 (0.57–1.81)	0.941		
**Had unprotected sex under the influence of alcohol in the past 6 months**				
No	1.0		1.0	
Yes	2.52 (1.14–4.46)	0.001	2.42 (1.18–4.96)	0.016
**Frequency of alcohol consumption**				
Non-frequent	1.0		1.0	
Frequent	2.87 (1.56–5.27)	0.001	2.06 (1.04–4.10)	0.038

**Notes:** Age at sexual debut was included as a continuous variable; CI *=* Confidence Interval; OR = Odds Ratio; aOR = Adjusted Odds ratio.

In weighted multivariate analysis, those who had sex with a male customer in the past 6 months (aOR = 7.34; 95%CI, 3.63–16.98, p<0.001), who had sex under the influence of alcohol in the past 6 months (aOR = 2.42; 95% CI, 1.18–4.96, p = 0.016) and “Frequent” alcohol consumers (aOR = 2.06; 95%CI, 1.04–4.10, p = 0.038) were more likely to have engaged in transactional sex in the past 12 months. Those who had their last sexual intercourse four weeks or more prior to the study (aOR = 0.26; 95% CI, 0.08–0.84, p = 0.024) were less likely to have engaged in transactional sex in the past 12 months.

## Discussion

This study was designed to test the hypothesis that FBWs are exposed to male customers and due to their limited financial means, some engage in transactional sex. Our findings also support the hypothesis that because these women promote the sale of alcoholic drinks, some of them consume alcohol which increase their likelihood of engaging in risky sexual behaviour and HIV and STI vulnerability. To our knowledge, this is the first comprehensive study conducted in SSA to have investigated HIV vulnerability and transactional sex among this subgroup of women.

This study found that FBWs earn low wages with a majority earning less than 50.000FCFA (the minimum wage in Cameroon as at August 2017 was 36,270 FCFA). Although monthly income was not statistically significantly associated with transactional sex, our results demonstrate that FBWs with a monthly income of less than 50,000 FCFA (< 89 US $) were more likely to have engaged in transactional sex (15.8% vs 12.9%) in the past 12 months. Over the years, there has been an increase in the cost of living in the city of Yaounde and some of these FBWs may have children, siblings and parents who depend on them for food and other livelihoods. Due to economic pressure and the need to care for dependents, these women have a likelihood of engaging in transactional sex to supplement their low income. The study found a marginally significant association between having other sources of income and engaging in transactional sex. The finding may suggest that transactional sex may have been one of the other sources from which these women made extra money.

In this study, 14.9% of FBWs indicated that they had engaged in transactional sex in the past 12 months. Additionally, 13.4% had sex with one or more of their male customers in the past 6 months. Similar findings have been reported in Cambodia [19] and Malawi [11] where FBWs were found to have sex with their male customers. In the Malawian study, FBWs indicated that money was the main reason why they sold sex to their male customers and some mentioned that without engaging in transactional sex, they will have nothing to eat [11]. In this study, it is important to keep in mind that these women may have engaged in transactional sex with either their male customers or with other sexual partners.

There was a strong positive association between having sex with a male customer and engaging in transactional sex which may suggest that the primary reason for having sex with male customers could have been for the exchange of money or gift [31, 32]. While some FBWs may be promoting the sale of beer, some may also be indirectly selling sex [33] to their male customers. This perhaps fits the categorization that some FBWs may be indirect sex workers as they supplement their income by selling sex on a regular basis or occasionally [19–21]. However, as reported in previous research, most women who engage in transactional sex would not identify themselves as sex workers [6, 16]. The above findings support the evidence that economic and food insecurity as well as survival needs may be motivating factors for engaging in transactional sex among women particularly those with dependents [4, 34, 35].

This study did not find any association between educational attainment, living arrangements and transactional sex among FBWs. Similar findings have been reported in a study conducted among a group of young women in Liberia [36]. Contrary to previous studies among women in the general population [16, 36], this study did not find any significant association between multiple sexual partners and engaging in transactional sex. Those who had their last sexual intercourse more than four weeks prior to the study were less likely to have engaged in transactional sex in the past 12 months. This may suggest that transactional sex was mainly carried out by FBWs who frequently had sexual intercourse. Nearly half (47.1%) of the FBWs in this study had sex within a week prior to the study. The explanation for this finding is unclear, but it is plausible that the existence of multiple and concurrent sexual partnership may be the reasons why most of the FBWs had their last sexual intercourse within a week.

This study found a positive association between alcohol consumption and engaging in transactional sex in the past 12 months. Participants who were “Frequent” alcohol consumers were more likely to have engaged in transactional sex. This finding is consistent with a previous study in Cambodia which reported that FBWs consume alcohol and some engaged in transactional sex [19]. Among women in the general population, similar findings in South Africa [37, 38] and Uganda [39] also reported that heavy alcohol consumption was associated with transactional sex. The above finding supports available evidence that alcohol consumption is associated with a number of risky sexual behaviours including transactional sex[25, 28], unprotected sex and inconsistent condom use [40, 41].

In the current study, about 17.6% of FBWs indicated that they had unprotected sex under the influence of alcohol in the past 6 months. Studies have reported that alcohol impairs cognitive functioning which leads to poor sexual decision-making [42, 43] and increases the risk of HIV and STI acquisition. Moreover, it is likely that some of these women may have had transactional sex with male partners who had consumed alcohol. Studies have reported that alcohol use is a risk factor for unprotected sex which increases the risk of HIV infection [44, 45].

The current study is of public health significance as it has identified some of the risky sexual practices among FBWs which increase their risk of acquiring HIV and STIs. The study demonstrates that FBWs represent a high-risk group for HIV and STI acquisition. The strength of this study relates to the fact that data were collected across all the seven administrative zones of Yaounde, and because the data were weighted, the findings could be generalised among FBWs in the city. Although the study findings are important to guide the design and implementation of HIV prevention programmes among FBWs, they must be interpreted in light of the following study limitations. Firstly, sensitive data (e.g., having sex with a male customer, number of life time sexual partners) were collected by self-report through face-to-face interviews, which may have resulted in under-reporting of risk behaviours due to social-desirability bias. Secondly, because the study was cross-sectional, it is not possible to draw any causal inferences. Thirdly, data were collected only during broad daylight and non-pick hours from the venues and due to safety and security reasons, data were not collected from nightclubs. Consequently, the study missed out some FBWs who work during peak hours and night shifts and their experience may have been different from those working during the day. Lastly, our study did not quantify alcohol consumption, as a result we recommend future studies to ask respondents about the quantity of alcohol they consume.

Despite these limitations, the current study has increased our knowledge and understanding of the risky behaviours that predispose FBWs to HIV and STI acquisition. It highlights the fact that FBWs are a subgroup of women at increased risk of HIV and STI infection. Future studies are needed to further understand the HIV and STI vulnerability among this group of women. A study to estimate the population size of FBWs would also be beneficial and epidemiological studies are needed to estimate the prevalence of HIV and STI among this subgroup of women.

## Conclusions

This study concludes that FBWs are a group of women whose profession exposes them to men and given that they promote beer consumption to male customers, most of these women also consume alcohol. These factors motivate them to engage in risky sexual practices which increases their risk of acquiring HIV and STI. Additionally, these women also earn low wages and due to limited socio-economic opportunities, some engage in transactional sex with their male customers as a means of survival. These findings are useful as they will inform the design and implementation of HIV prevention interventions to target this group of women. Although interventions aimed towards reducing risky sexual behavior are important, there is a need for economic empowerment interventions directed towards these women. Such interventions will enable them to be financially viable which may decrease their likelihood of engaging in transactional sex and hence reducing their HIV and STI vulnerability.

## Supporting information

S1 Questionnaire This is the questionnaire that was used to collect data for the study. (DOCX)

S1 Study Dataset This is the study dataset. (XLS)
