# Supplementary material for: Socio-economic vulnerabilities and HIV: Drivers of transactional sex among female bar workers in Yaoundé, Cameroon
Source: PLoS One. 2018 Jun 18;13(6):e0198853. doi: 10.1371/journal.pone.0198853 (PMC6005536; doi:10.1371/journal.pone.0198853)
Supplement: S1 Questionnaire — (DOCX) [file pone.0198853.s001.docx]

**QUESTIONNAIRE**

**HIV Vulnerability, Sexual Harassment and its Impacts on Mental Health among Female Bar Workers in Yaoundé, Cameroon**

| Administrative District: | Quarter/Neighborhood: |
| --- | --- |
| Interviewer Name: | Supervisors Name: |
| Interviewer’s Number: | Supervisor’s Number: |
| Date of interview: | Respondent/Participant ID: |

**Introduction:** Hello, my name is (-------) and I am working for the **Community Research & Training Institute**. We are working on a project concerned with HIV, sexual harassment and mental health among young women who promote and/or sell beer in bars, restaurants and beer parlours. The interview will take about 20-25 minutes. All information we collect will remain confidential and your answers will never be shared with anyone than our project research team.

I will read you the information sheet which will provide you with more information about the research. When you have any questions please feel free to ask me.

[Interviewer reads Participant Information Sheet to the respondent and ask respondent if she has any questions]

MAY I START NOW?

- Yes, permission is given: Provide informed consent form for signing and then begin the interview.
- No, permission is not given, Move on to a different respondent.

**SECTION A. SOCIO-DEMOGRAPHIC CHARACTERISTICS**

| **NO.** | **QUESTIONS** | **CODING CATEGORIES** | **SKIP PATTERNS** |
| --- | --- | --- | --- |
|  | **INTERVIEWER:**  Thank you for your interest in this study. I would start by asking you some questions about yourself. Let me assure you that all responses you provide will be treated with privacy and confidentiality. Your answers will not be told to any one aside from the research team. | | |
| A1. | How old are you? / How old were you at your last birthday? | Age (in completed years)__ __ |  |
| A2. | What is your current marital status? | ❒_1_Never Married  ❒_2_Currently Married  ❒_3_Divorced/Separated/Widowed |  |
| A3. | Which of the following best describes your level of education? | ❒_1_Completed primary school  ❒_2_Completed secondary school  ❒_3_Completed high school  ❒_4_University degree and above |  |
| A4. | How much do you earn a month as a beer promotion worker? | ❒_1_Less than 49,999FCFA  ❒_2_50,000FCFA+ |  |
| A5. | Do you have other sources of income apart from the work that you are currently doing? | ❒_1_No ❒_2_Yes |  |
| A6. | Which of the following best describes your current living arrangements? | ❒_1_Live alone  ❒_2_Live with someone | **LIVE ALONE🡪 A8**  **LIVE WITH SOMEONE🡪A7** |
| A7. | How many individuals do you current live with? | ❒_1_One to Three  ❒_2_Four to Six  ❒_3_More than Seven |  |
| A8. | For how long have you been working as a beer promotion girl? | ❒_1_ 3-12 months  ❒_2_ 12 months+ |  |

**SECTION B. SEXUAL BEHAVIOUR AND VULNERABILITY**

| **NO.** | **QUESTIONS** | **CODING CATEGORIES** | **SKIP PATTERNS** |
| --- | --- | --- | --- |
| **INTERVIEWER:**  Now, I would like to ask you some questions about sexual activity in order to gain a better understanding of some important life issues. I would be happy if you respond as truthfully as you can to the questions. The information you provide will remain strictly confidential and will not be told to anyone. | | | |
| B1. | Women usually have sex with different men over their lifetime. In total, with how many different men have you had sexual intercourse in your lifetime?  [ Please give your best guess] | ❒_1_ Less than 10 men  ❒_2_10-19 men  ❒_3_ 20 or more men |  |
| B2. | How old were you when you had your first sexual intercourse?  [Please give your best guess] | ____________(years) |  |
| B3. | How many sexual partners do you currently have? | ❒_1_ None  ❒_2_ One only  ❒_3_ Two to three  ❒_4_ Four or more |  |
| B4. | In the past 12 months, have you ever had sex in exchange for money or gift? | ❒_1_ No  ❒_2_ Yes |  |
| B5. | In the past **6 months**, have you had sex with a male customer? | ❒_1_ No  ❒_2_ Yes | **NO 🡪B9**  **YES 🡪B6** |
| B6. | How many male customers did you have sex with in the past 6 months? | ❒_1_ One only  ❒_2_ Two or more |  |
| B7. | Did you enter into a sexual relationship with the male customer? | ❒_1_ No ❒_2_ Yes | **No 🡪B9**  **YES 🡪B8** |
| B8. | How long did the relationship last? | ❒_1_Less than 3 months  ❒_2_Four months or more |  |
| B9. | When was the last time you had sexual intercourse? | ❒_1_Less than a week ago  ❒_2_One to two weeks ago  ❒_3_Three to four weeks ago  ❒_4_Five weeks or more ago. |  |
| B10. | The last time you had sexual intercourse, was a condom used? | ❒_1_No ❒_2_Yes |  |
| B11. | Now, I would like to ask you some questions about drinking alcohol. Do you drink/ consume alcohol? | ❒_1_ No ❒_2_ Yes | **No🡪SECTION C**  **YES 🡪B17** |
| B12. | During the past one month, on how many days did you have at least one drink of alcohol | ❒_1_1 or 2 days  ❒_2_3 to 5 days  ❒_3_6 to 9 days  ❒_4_10 to 19 days  ❒_5_20 to 29 days  ❒_6_All 30 days |  |
| B13. | In the past 6 months, did you have unprotected sex while under the influence of alcohol? | ❒_1_ No ❒_2_ Yes |  |

**SECTION C: HIV AWARENESS, STI SYMPTOMS AND TREATMENT**

| **NO.** | **QUESTIONS** | **CODING CATEGORIES** | **SKIP PATTERNS** |
| --- | --- | --- | --- |
| **INTERVIEWER:**  Now I would like to ask you some questions related to HIV testing and symptoms related to other sexually transmitted infections. I am assuring you once more that your answers are completely confidential and will not be told to anyone**.** | | | |
| C1. | I don’t want to know the results, but have you ever had an HIV test? | ❒_1_No  ❒_2_Yes | **No🡪C2**  **YES 🡪C3** |
| C2. | Why have you not had an HIV test?  (Read responses, probe and ask for other reasons) | ❒_1_ I did not feel ready to do a test  ❒_2_ I felt that I was not infected  ❒_3_ I think I am not at risk of getting HIV  ❒_4_ Afraid to discover a positive HIV test  ❒_5_ Lack of time  ❒_6_Others(Specify)____________ |  |
| C3. | When was the last time you had an HIV test? | ❒_1_ Less than 6 months ago  ❒_2_ Six to 12 months  ❒_3_ 13 months or more ago |  |
| C4. | Which of the following symptoms have you had in the past 12 months (Please tick all that apply)  ❒Lower abdominal pain  ❒Vaginal discharge  ❒Itching around genital area  ❒Pain or burning sensation when urinating  ❒Others (Please specify)______________ | | |
| C5. | In the past 12 months, have you been diagnosed of any sexually transmitted infection (STI)?  ❒_1_No ❒_2_ Yes | | **No🡪SECTION D**  **YES 🡪C6** |
| C6. | Which of the following sexually transmitted infections were you diagnosed of?  ❒_1_Chlamydia  ❒_2_Gonnorrhoea  ❒_3_Syphilis  ❒_4_Others (Specify)________________ | | |
| C7. | Where did you receive treatment?  (Probe and ask for other) | ❒_1_ Hospital  ❒_2_ Health Centre  ❒_3_ Private Clinic  ❒_4_ Seek assistance from pharmacy  ❒_5_ Traditional medication  ❒_6_Others (Specify) |  |

**SECTION D. WORKPLACE SEXUAL HARRASSMENT**

| **NO.** | **QUESTIONS** | **CODING CATEGORIES** | **SKIP PATTERNS** |
| --- | --- | --- | --- |
| **INTERVIEWER:**  Thank you so much for your continuous interest in this study. Now, I would like to ask you certain questions which are related to sexual harassment. I would start by explaining the definition of sexual harassment to you and then ask you some questions. | | | |
| ““Sexual harassment is any unwelcome sexual advance, unwelcome request for sexual favour, verbal or physical conduct or gesture of a sexual nature, or any other behaviour of a sexual nature that might reasonably be expected or be perceived to cause offence, humiliation or intimidation to the person.” | | | |
| D1. | In the past 3 months, which of the following unwelcome behaviours have you experienced from male customers at your workplace?  ❒_1_Touched any part of your body like buttocks  ❒_2_Sexually suggestive comments or jokes that made you feel offended  ❒_3_Sexual advances including providing their telephone numbers to contact them  ❒_4_Inappropriate starring that made you feel uncomfortable  ❒_5_Intrusive questions about your private life or physical appearance  ❒_7_Inappropriate physical contact or forcibly kissing  ❒_8_Repeated demands for a date despite your rejection  ❒_9_Others (Specify)_______________ | | |
| D2. | In the past month, have you been verbally abused/insulted by a male customer? | ❒_1_No ❒_2_Yes | **No🡪D4**  **YES 🡪D3** |
| D3. | How often do you experience verbal abuse/insults from male customers? | ❒_1_Not so often  ❒_2_ Somewhat often  ❒_3_Very often  ❒_4_Extremely often |  |
| D4. | When was the last time you experienced sexual harassment? | ❒_1_ Less than month ago  ❒_2_ One to three months ago |  |
| D5. | How did you feel after you experienced the sexual harassment from the male client? | ❒_1_Offended  ❒_2_ Humiliated  ❒_3_ Threatened  ❒_4_ Uncomfortable  ❒_5_Afraid  ❒_6_Feel Normal  ❒_7_Others(Specify) |  |
| D6. | To whom did you seek support/advice from after experiencing your last sexual harassment? | ❒_1_Did not request for support  ❒_2_ Manager/Supervisor  ❒_3_ Boss  ❒_4_ Co-worker  ❒_5_ Family member  ❒_6_ Police  ❒_7_ Others Specify)__________ |  |

**SECTION E: MENTAL HEALTH OUTCOMES**

| **INTERVIEWER:**  I would now be asking you some questions related to your mental health. I am going to read some statements which may indicate how much the statement applied to you in the past 4 weeks. Remember, there are no right or wrong answers | | | | | | | |
| --- | --- | --- | --- | --- | --- | --- | --- |
|  |  | All of the time(1) | Most of the time(2) | A good bit of the time(3) | Some of the time(4) | A little of the time(5) | None of the time(6) |
| E1. | You have felt so depressed that nothing could cheer you up | ❒_1_ | ❒_2_ | ❒_3_ | ❒_4_ | ❒_5_ | ❒_6_ |
| E2. | You have felt so discouraged and been in low spirits | ❒_1_ | ❒_2_ | ❒_3_ | ❒_4_ | ❒_5_ | ❒_6_ |
| E.3. | You have been a happy person | ❒_1_ | ❒_2_ | ❒_3_ | ❒_4_ | ❒_5_ | ❒_6_ |
| E.4. | You have been a very nervous person | ❒_1_ | ❒_2_ | ❒_3_ | ❒_4_ | ❒_5_ | ❒_6_ |
| E5. | ‘Have you felt calm and peaceful?’ | ❒_1_ | ❒_2_ | ❒_3_ | ❒_4_ | ❒_5_ | ❒_6_ |

**Thank you for taking the time to participate in this survey. Your responses will be very helpful in answering the research questions which may lead to awareness raising and improvement of your working conditions.**
